# Integrating the Epigenome and Transcriptome of Hepatocellular Carcinoma to Identify Systematic Enhancer Aberrations and Establish an Aberrant Enhancer-Related Prognostic Signature

**DOI:** 10.3389/fcell.2022.827657

**Published:** 2022-03-01

**Authors:** Peng Huang, Bin Zhang, Junsheng Zhao, Ming D. Li

**Affiliations:** ^1^ State Key Laboratory for Diagnosis and Treatment of Infectious Diseases, National Clinical Research Center for Infectious Diseases, Collaborative Innovation Center for Diagnosis and Treatment of Infectious Diseases, The First Affiliated Hospital, Zhejiang University School of Medicine, Hangzhou, China; ^2^ Research Center for Air Pollution and Health, Zhejiang University, Hangzhou, China

**Keywords:** enhancer, super-enhancer, DNA methylation, histone modification, prognostic model, eRNA, RNA-seq, hepatocellular carcinoma

## Abstract

Recently, emerging evidence has indicated that aberrant enhancers, especially super-enhancers, play pivotal roles in the transcriptional reprogramming of multiple cancers, including hepatocellular carcinoma (HCC). In this study, we performed integrative analyses of ChIP-seq, RNA-seq, and whole-genome bisulfite sequencing (WGBS) data to identify intergenic differentially expressed enhancers (DEEs) and genic differentially methylated enhancers (DMEs), along with their associated differentially expressed genes (DEE/DME-DEGs), both of which were also identified in independent cohorts and further confirmed by HiC data. Functional enrichment and prognostic model construction were conducted to explore the functions and clinical significance of the identified enhancer aberrations. We identified a total of 2,051 aberrant enhancer-associated DEGs (AE-DEGs), which were highly concurrent in multiple HCC datasets. The enrichment results indicated the significant overrepresentations of crucial biological processes and pathways implicated in cancer among these AE-DEGs. A six AE-DEG-based prognostic signature, whose ability to predict the overall survival of HCC was superior to that of both clinical phenotypes and previously published similar prognostic signatures, was established and validated in TCGA-LIHC and ICGC-LIRI cohorts, respectively. In summary, our integrative analysis depicted a landscape of aberrant enhancers and associated transcriptional dysregulation in HCC and established an aberrant enhancer-derived prognostic signature with excellent predictive accuracy, which might be beneficial for the future development of epigenetic therapy for HCC.

## Introduction

Liver cancer is the sixth most common malignant tumor and the third leading cause of cancer-related deaths, accounting for approximately 700,000 deaths annually worldwide and poses a severe health threat and economic burden to the world (Likhitsup and Parikh, 2020; Sung et al., 2021). This is especially true in China, which has the largest HCC risk population (HBV carriers) throughout the world. The latest epidemiological report showed that primary liver cancer is the fourth most common tumor in China (Feng et al., 2019) and the vast majority of liver cancers are HCCs. Since there are usually no evident symptoms in the early developmental state of liver cancer, patients are often diagnosed in the late stage of liver cancer, resulting in an extremely high probability of death (Grandhi et al., 2016). Although the survival duration of early- and intermediate-stage HCCs has improved over the past decades, the prognosis for advanced-stage HCC patients has remained poor, with no significant improvement. Even with the survival benefits of several first‐ and second‐line therapeutic options available for patients with advanced HCC, such as sorafenib and lenvatinib, the median survival time of intermediate to advanced HCC is only 1–2 years (Marrero et al., 2018). Clinical studies of immune checkpoint inhibitors have yielded promising survival benefits, although the suppressive milieu and tumor immunosurveillance escape mechanisms in the liver still dampen the effectiveness of immunotherapy (Nakano et al., 2020). Hence, there is an urgent need to explore the underlying genetic and epigenetic mechanisms implicated in hepatocarcinogenesis to identify potential targets/biomarkers for the diagnosis, treatment and prognosis of HCC.

Cancer is a complex disease involving both genetic mutations and epigenetic aberrations. By definition, epigenetics refers to heritable states of gene activities that do not involve alteration of DNA sequence itself. Epigenetic changes such as DNA hypermethylation or hypomethylation, dysregulation of histone modification patterns, chromatin remodeling, and aberrant expression of noncoding RNAs are demonstrated to be involved in the initiation and progression of HCC (Wahid et al., 2017). Unlike genetic mutations, epigenetic alterations are reversible and various drugs targeting epigenetic regulators have exhibited viable therapeutic potential for solid tumors in both preclinical and clinical studies (Cheng et al., 2019). A better understanding of the epigenetic mechanisms underlying hepatocarcinogenesis will facilitate the discovery of new targets and biomarkers for HCC therapy.

Like most malignancies, HCC is also characterized by widespread abnormal gene expression. Enhancers are distal, noncoding genomic regulatory elements with multiple transcription factor binding sites that interact with promoters to enhance the transcription of target genes. Nucleosomes in the neighborhood of active enhancers usually contain histones with iconic posttranslational modifications, such as H3 lysine monomethylation (H3K4me1) and H3 lysine acetylation (H3K27ac) at their amino termini (Shlyueva et al., 2014). Super-enhancers are large clusters of enhancers that synergistically promote gene transcription (Herranz et al., 2014). Emerging evidence shows that cancer cells can acquire super-enhancers in the vicinity of key oncogenes, such as *MYC* and *TAL1*, during the development of cancer (Hnisz et al., 2013; Herranz et al., 2014; Mansour et al., 2014). Moreover, pancancer studies of TCGA data also showed wide-spread aberrant super-enhancer activities in cancers (Chen et al., 2018a; Chen and Liang, 2020).

In HCC, Wong et al. demonstrated that the super-enhancer landscape and components of the *trans*-acting super-enhancer complex, composed of *CDK7*, *BRD4*, *EP300*, and *MED1*, were significantly altered (Tsang et al., 2019). Additionally, Deng et al. reported an aberrant landscape of active enhancers developed in cirrhosis and conserved in hepatocarcinogenesis (Yang et al., 2020). However, those two studies lacked a comprehensive collection of enhancers in the liver, reliable identification of enhancer target genes, and replication of enhancer aberrations in independent cohorts.

In the present study, through the integration of transcriptome and epigenome data, we aimed to: 1) manually curate a comprehensive catalog of enhancers in the liver; 2) systematically identify and replicate enhancer aberrations and associated target genes in HCC; and 3) explore the function and prognostic significance of identified aberrant enhancers.

## Materials and Methods

### Patient Data and Tissues Collection

Paired tumor tissues and adjacent non-tumor tissues used in this study were collected from 33 HCC patients who underwent hepatectomy at the First Affiliated Hospital, Zhejiang University School of Medicine. Board-certified pathologists reviewed each specimen to confirm that all frozen sections were histologically consistent with tumor or non-tumor tissues. This study was approved by the Institutional Review Board of The First Affiliated Hospital. Written informed consent was obtained from each participant.

### High-Throughput Sequencing and Computational Preprocessing

DNA methylation and gene expression of 33 pairs of tumour and adjacent tissues were assessed by whole-genome bisulfite sequencing (WGBS) and mRNA-seq on the Illumina X Ten platform with standard procedures. After quality control, clean WGBS reads were aligned with the reference genome (hg38) using Bismark (v. 0.16.1) (Krueger and Andrews, 2011) with default parameters. The harvested count data for each strand were combined for methylation level estimation. Differentially methylated loci (DML) and differentially methylated regions (DMRs) were detected with customized R scripts like our previous WGBS study (Huang et al., 2021). For RNAseq data, clean reads that passed quality control were aligned with the hg38 genome, and the reference transcriptome was downloaded from GENCODE (v. 29) (Harrow et al., 2012) with STAR (v. 2.5.2a) (Dobin et al., 2013). Estimated raw count gene expression from STAR was imported into DESeq2 (Love et al., 2014) for differential expression analysis. STAR generated alignment BAM files were utilized as input for enhancer RNA (eRNA) expression quantification via bedtools (v. 2.27.1) (Quinlan and Hall, 2010). More details about high-throughput sequencing and bioinformatic preprocessing can be found in the Supplementary Methods.

### Curation of a Comprehensive Catalog of Enhancers in Liver

Eleven histone ChIP-seq liver relevant samples were collected from the public domain. Specifically, bed files containing the pseudo-replicated peaks identified from six H3K4me1- and H3K27ac-based ChIPseq profiled samples (i.e., one HepG2, one Hepatocyte, and four normal adult liver tissue samples) were downloaded from ENCODE (Consortium, 2004). For each ENCODE sample, regions with overlapped H3K27ac and H3K4me1 peaks were annotated as active enhancers, and regions with only H3K4me1 peaks were considered as primed enhancers. In one case of an adult liver ENCODE sample without H3K27ac profiling data, H3K4me1 peaks were included as enhancers (primed or active). Four types of histone (including H3K4me1 and H3K27ac) ChIP-seq profiling-based ChromHMM state annotation files of five adult liver tissue samples (i.e., one normal liver sample and two tumor and matched adjacent cirrhosis samples from two HCC patients) were retrieved from the recent integrative epigenomic study on HCC (Hlady et al., 2019). Specifically, regions whose ChromHMM states were annotated as “poised enhancer” (refers to regions with only H3K4me1 peaks) were included as primed enhancers, and regions annotated as “active enhancer” (refer to regions with both H3K4me1 and H3K27ac) were collected as active enhancers. All (active or primed) enhancers from each sample were merged together via bedtools (Quinlan and Hall, 2010). Afterwards, enhancers with a lenth of <50 bp or overlapped with any promoter (upstream 1,500 bp to downstream 500 bp from TSS) were excluded from further analysis. The concurrence of each merged enhancer was estimated as the number of ChIP-seq samples in which the merged enhancer was annotated as a primed or active enhancer. In other words, a merged enhancer with higher concurrence represents a more highly conserved and reliable enhancer among those 11 liver-related ChIP-seq samples.

### Identification of Intergenic Differentially Expressed Enhancers and Associated Differentially Expressed Genes

The collected enhancers in the liver were divided into two groups, namely, intergenic enhancers and genic enhancers, according to their genomic locations. For intergenic enhancers, the read count-based expression levels of eRNAs were estimated via the “coverage” module of bedtools (Quinlan and Hall, 2010). A paired *t*-test was applied to the normalized expression (log_2_ transformed fragment per million, log_2_ FPM) of each eRNA to identify significant differentially expressed eRNA (|log_2_ fold change of FPM| > 0.5 and BH-FDR < 0.05). Intergenic enhancers with significant differential expression of eRNA were defined as intergenic differentially expressed enhancers (intergenic DEEs). Nearby (TSS located ± 1 Mb from the center of corresponding intergenic DEEs) differentially expressed genes (DEG) (|log_2_ fold change (LFC)|> 0.5 and BH-FDR < 0.05) displayed a significant correlation (Spearman Rho ≥ 0.7 and Bonferroni-corrected *p*-value < 0.01) with eRNA expression were identified as intergenic DEE-associated DEGs (intergenic DEE-DEGs).

### Replication of Intergenic DEE-DEGs

For independent replication of intergenic DEE-DEGs, four HCC RNA-seq datasets were downloaded from the GEO: GSE77314 (paired tumor and adjacent nontumor tissue samples from 50 HCC patients) (Liu et al., 2016), GSE124535 (paired tumor and adjacent nontumor tissue samples from 35 HCC patients) (Jiang et al., 2019), GSE148355 (62 tumor and 47 adjacent nontumor samples) (Yoon et al., 2021), and GSE77509 (paired tumor and adjacent nontumor samples from 20 HCC patients) (Yang et al., 2017). The same protocols in the discovery cohort were applied to detect intergenic DEEs and associated DEE-DEGs in these four datasets. Afterward, identified intergenic DEEs and DEE-DEGs from each dataset were compared with those from the discovery cohort to calculate the concurrence of each intergenic DEE and DEE-DEG. Specifically, the concurrence of each DEE was calculated as one plus the number of GEO datasets in which the DEE was successfully replicated, while the concurrence of each DEE-DEG was calculated as one plus the number of GEO datasets in which the corresponding DEE and DEG were significant and the correlation between them was also significant.

### Assessment of the Roles of Epigenetic Modification Aberrations in Intergenic DEEs

Intergenic DEEs that overlapped with at least one DMR and displayed significant methylation-eRNA Spearman correlation (BH-FDR < 0.05) were defined as methylation-associated DEEs, and corresponding DEE-DEGs were classified as methylation-associated DEE-DEGs. Meanwhile, we further investigated the dysregulation of histone posttranslational modification (PTM) modifiers and their potential implications in those identified intergenic enhancer aberrations. Top differentially expressed histone PTM modifiers (|LFC| > 1 and BH-FDR < 5%) in the discovery cohort were screened out for subsequent coexpression analyses to determine the ratios of DEEs and DEE-DEGs that significantly correlated (|Spearman correlation coefficient| > 0.5 and BH-FDR < 5%) with the mRNA expression of those histone PTM modifiers.

### Identification of Genic Differentially Methylated Enhancers and Associated Differentially Expressed Genes

Reliable genic enhancers (concurrence among the 11 ChIP-seq samples ≥2) that overlapped (length_overlap_ ≥ 200 bp, length_overlap_/length_enhancer_ ≥ 0.3, and with at least 5 CpGs) with at least one DMR were identified as potential genic differentially methylated enhancers (genic DMEs). For each potential genic DME, their associated DEGs were screened *via* the Spearman correlation test. Nearby (distance of enhancer to TSS ≤ ± 1 Mb) DEGs (|LFC|> 0.5 and BH-FDR < 0.05) that show significant correlation (|Rho| ≥ 0.5 and FDR ≤ 0.01) between gene expression and DNA methylation level were identified as genic DEE-associated DEGs (genic DME-DEGs). Genic DME candidates with at least one associated DEG were identified as genic DMEs.

### Replication of Genic DMEs and DME-DEGs

The normalized gene expression and DNA methylation level matrix of TCGA-LIHC were retrieved *via* the RTCGA R package (Kosinski and Biecek, 2015). For each genic DEE-DEG pair identified in the discovery cohort, we examined the significance of differential methylation, differential expression, and Spearman correlation between DNA methylation and gene expression in TCGA-LIHC. A genic DEE-DEG pair was considered as “successful replication” only when there was simultaneous significant differential methylation, differential expression, and a significant correlation between methylation and expression in TCGA-LIHC. Considering the platform limitation of the 450 k methylation array in covering enhancer CpG, we classified all replication failures of DEE-DEGs into two groups: 1) “type I failure” refers to replication failure due to the lack of CpG for corresponding genic DMEs in TCGA-LIHC, and 2) “type II failure” refers to replication failure except type I failure. The raw replication rate of genic DEE-DEGs was calculated as the ratio of genic DEE-DEGs that achieved successful replication, while the platform-adjusted replication rate was defined as: Count_Successful replication_/(Count_Successful replication_ + Count_Type II failure_)*100.

### Functional Enrichment of Aberrant Enhancer-Associated Differentially Expressed Genes

AE-DEGs were defined as the union of those identified intergenic DEE-DEGs and genic DME-DEGs. Pathway/gene ontology (GO) enrichment analyses of upregulated and downregulated AE-DEGs were performed *via* the online web tool Metascape (Zhou et al., 2019). In addition, 10 cancer hallmark gene sets were downloaded from the Cancer Hallmark Gene (CHG) database (Zhang et al., 2020a). The enrichment degrees of AE-DEGs for cancer hallmarks were evaluated through a hypergeometric test followed by BH-FDR multitest correction in R.

### Bioinformatic Confirmation of AE-DEGs Using Public Hi-C Data

The bed files containing topologically associated domains (TADs) and chromatin loops of Hi-C-profiled HepG2 and one normal adult liver tissue sample were downloaded from the 3D Genome Browser (http://3dgenome.fsm.northwestern.edu/) (Wang et al., 2018). Each pair of AE and AE-DEG was examined to determine whether both the enhancer and its associated DEG were located in the same TAD or located in the two elements of a chromatin loop, respectively.

### Establishment of an AE-Derived Prognostic Model

Clinical phenotype data, including overall survival (OS) time and status, were retrieved from the integrated TCGA pancancer clinical data resource (Liu et al., 2018a). Univariate Cox proportional hazards regression analysis was conducted to screen for AE-DEGs associated with the OS of HCC patients in TCGA-LIHC via the function “coxph” in the R package “survival” (Therneau, 2020). AE-DEGs with univariate Cox *p*-value < 0.05 were incorprated into the least absolute shrinkage and selection operator (LASSO) regression model by using the glmnet package (Friedman et al., 2010) for identification of the most prominent survival-associated AE-DEGs in TCGA-LIHC. Afterward, the multivariate proportional hazards Cox regression model was employed to establish a gene signature for predicting the OS of HCC patients. Multivariate Cox regression-derived coefficients (β) were used to calculate the risk score as follows: risk score = (β_gene1_ * normalized expression level of gene1 + β_gene2_ * normalized expression level of gene2 + … + β_geneN_ * normalized expression of geneN) (Lossos et al., 2004). Based on the optimal cutoff of risk score determined by minimizing log-rank test *p*-value, HCC patients were divided into high- and low-risk groups, whose differences in OS probability across time were visualized through a Kaplan-Meier survival curve by using the function “ggsurvplot” in the survminer package (Alboukadel Kassambara, 2021). The prognostic performance of the risk score was evaluated by time-dependent receiver operating characteristic (ROC) curve analysis via the function “survivalROC” in the survivalROC package (Patrick, 2013). The independent prognostic role of the identified gene signature in TCGA-LIHC was assessed by building a multivariate Cox regression model including the risk group, age, gender, and pathologic tumor-node-metastasis (TNM) stage of each patient. All factors that passed through the multivariate Cox regression model were utilized for the construction of a predictive nomogram *via* the rms package (RMS, 2021). Calibration plots and time-dependent ROC curves were applied to assess the predictive performance of the established nomogram.

### Validtion of the AE-Derived Prognostic Model

Regarding the independent validation of the prognostic signature, clinical phenotypes and gene expression data of the International Cancer Genome Consortium Liver Cancer-RIKEN (LIRI-JP) were downloaded from the ICGC website. Multivariate Cox regression-derived coefficients from TCGA-LIHC were used to calculate the corresponding risk score for each patient in ICGC-LIRI. Similarly, ICGC-LIRI patients were divided into high- and low-risk groups according to the cutoff determined by minimizing the log-rank test *p*-value. Comparison of the difference in OS probability, evaluation of predictive performance, and assessment of predictive independence were performed with identical procedures employed for TCGA-LIHC.

## Results

### Comprehensive Collection of Enhancers in the Liver

**FIGURE 1:**
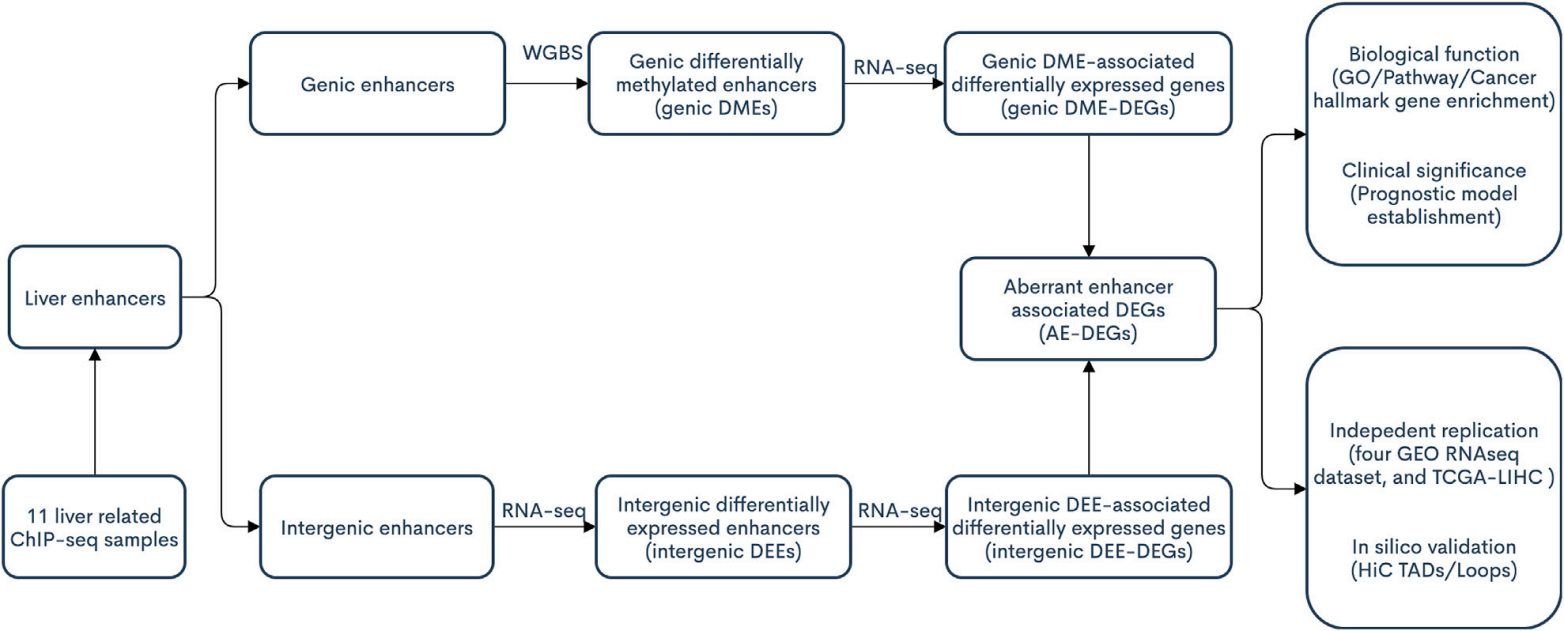
The schematic flowchart of the present study.

**FIGURE 2:**
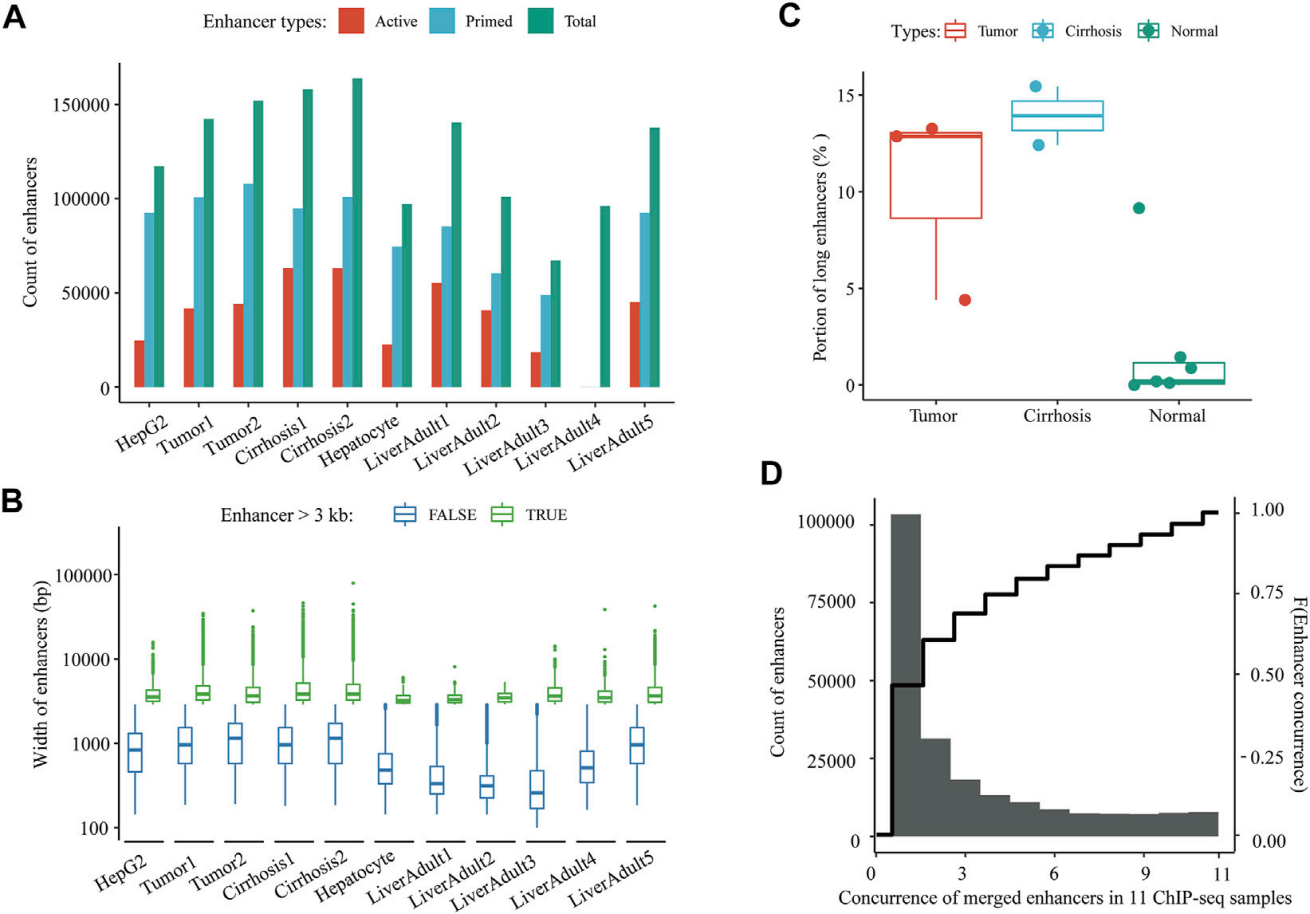
A comprehensive catalog of enhancers in the liver. **(A)** Count of active and primed enhancers in each liver-relevant ChIP-seq sample. **(B)** Length distribution of enhancers in each liver-relevant ChIP-seq sample. **(C)** Proportions of long enhancers in three types of liver-relevant ChIP-seq samples. And **(D)** Distribution of the concurrence of all merged enhancers among 11 liver-relevant ChIP-seq samples.

The procedures of this study are shown in the schematic flowchart (Figure 1). Among 11 liver-relevant ChIP-seq profiled samples, we identified numerous enhancers whose counts ranged from 96,124 to 163,953 (Figure 2A; Table S1.1). On average, there were 124,838 enhancers in each sample, among which approximately one third (32.08%) were active enhancers with both H3K4me1 and H3K27ac peak signals (Table 1; Supplementary Table S1.1). Interestingly, there was a higher proportion of long enhancers (length > 3 kb) in nonnormal samples (i.e., tumoral and cirrhosis samples), and the median widths of classical enhancers (length ≤ 3 kb) were also higher than those of normal samples (Figures 2B,C; Supplementary Table S1.2). Specifically, the mean percentages of long enhancers (>3 kb) in tumor samples, adjacent cirrhosis samples, and normal liver samples were 10.17, 12.93, and 1.97%, respectively (Figure 2C). After combining these enormous enhancers, we obtained a comprehensive catalog of 223,007 unique enhancers in the liver. Over one half (53.64%) of them were concurrent enhancers that consistently existed in at least two samples (Figure 2D; Supplementary Table S1.3). In addition, genomic location-based annotation showed that approximately 29.84% (66,551/223,007) of those 223,007 enhancers were located in intergenic regions (Supplementary Table S1.3).

**TABLE 1:** Characteristics and enhancer identification strategies applied for 11 liver-relevant ChIP-seq samples.

Sample name	Source	H3K4me1	H3K27ac	Enhancer identification strategy
HepG2	ENCODE (Consortium, 2004)	√	√	H3K4me1 only (primed enhancer) + H3K4me1 and H3K27ac (active enhancer)
Hepatocyte		√	√	
LiverAdult 1		√	√	
LiverAdult 2		√	√	
LiverAdult 3		√	√	
LiverAdult 4		√	X	H3K4me1 (primed or active enhancer)
LiverAdult 5	The integrative epigenomic HCC study (Hlady et al., 2019)	√	√	H3K4me1 only (primed enhancer) + H3K4me1 and H3K27ac (active enhancer)
Cirrhosis 1		√	√	
Cirrhosis 2		√	√	
Tumor 1		√	√	
Tumor 2		√	√	

### Activated and Repressed Intergenic Enhancers Show Different Patterns in Concurrence and Transcriptional Regulation in HCC

**FIGURE 3:**
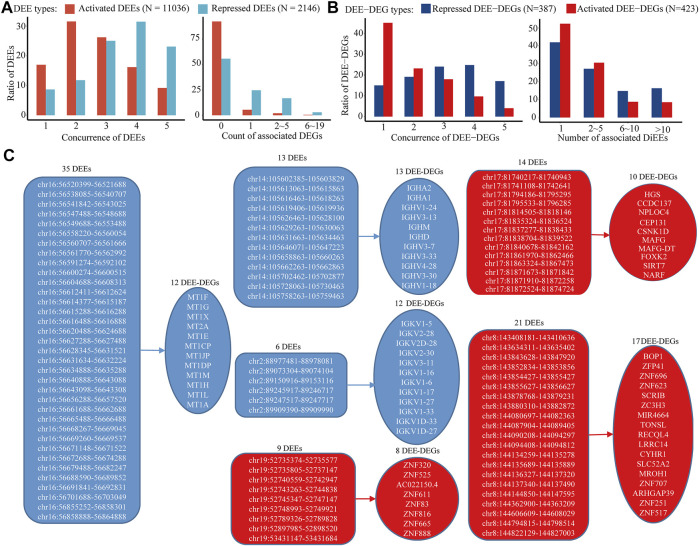
Distinct patterns of activated and repressed intergenic enhancers in HCC. **(A)** (left) Distribution of the concurrence of identified intergenic DEEs among five RNA-seq datasets and (right) distribution of the number of associated intergenic DEE-DEG of each DEE. **(B)** (left) Distribution of the concurrence of identified intergenic DEE-DEGs among five RNA-seq datasets and (right) distribution of the number of associated DEE of each DEE-DEG. **(C)** Gene clusters associated with aberrant super-enhancers. Only gene clusters with at least five DEE-DEGs and super-enhancers with at least five DEEs were displayed. Activated DEEs and DEE-DEGs are shown in red color, and repressed DEEs and DEE-DEGs are shown in blue.

In the discovery cohort, 23,601 of the 66,551 collected intergenic enhancers displayed active transcription of eRNA, and 13,182 of them were identified as intergenic DEEs, including 11,036 activated DEEs and 2,146 repressed DEEs (Figure 3A). Through bioinformatic inferrence for target genes, 842 activated DEEs and 951 repressed DEEs were found to be correlated with 423 upregulated DEE-DEGs, and 387 downregulated DEE-DEGs, respectively (Figure 3B; Supplementary Table S2). Although the number of activated DEEs was over fivefold that of repressed DEEs (11036 *vs.* 2,146), each repressed DEE was found to be simultaneously associated with more DEE-DEGs (Figure 3A). Specifically, 19.67% of repressed DEEs displayed high correlations with multiple DEE-DEGs, while only 2.28% of activated DEEs showed this pattern (Figure 3A). Moreover, 387 downregulated and 423 upregulated DEE-DEGs also showed differences in terms of the number of associated DEEs. Compared with upregulated DEE-DEG, each downregulated DEE-DEG tended to be simultaneously regulated by more DEEs (Figure 3B). Taken together, we found a higher portion of potential transcriptional master regulators among the repressed DEEs, and more downregulated DEE-DEGs were simultaneously associated with aberrant super-enhancers that were consisted of multiple adjacent synergistic enhancers (Figure 3C). For example, in 16q13, a cluster of 35 repressed intergenic DEEs was identified as potential regulators of the metallothionein (MT) family (i.e., each of the 35 DEEs was significantly correlated with the expressions of all 12 metallothionein genes) (Figure 3C; Table 2). A literature searching revealed that nine of those 12 MT genes were previously implicated in HCC (Table 2). Besides, in chromosome 17, we also identified a super-enhancer whose activation was correlated with upregulation of 10 DEGs including nine previously-reported oncogenes in HCC or other cancers (Table 2 **)**. Beyond these, we also identified another four gene clusters likely regulated by super-enhancers on chromosomes 14, 2, 19, and 8 (Figure 3C).

**TABLE 2:** Summary information about two representative sets of DEE-DEGs regulated by intergenic DEE clusters.

Enhancer cluster	Average LFC.DEE	Avergae LogFDR.DEE	Gene name	LFC DEG	FDR DEG	Average rho	Average concurrence	Implicated cancers
chr16:56520399–56864888 (∼344 kb, 35 DEEs)	−3.3	6.62	*MT1A*	−**2.08**	**1.45E-06**	**0.76**	**2.56**	HCC (Ning et al., 2021)
			*MT1CP*	−2.01	1.20E-03	0.82	3.51	Unknown
			*MT1DP*	−**1.37**	**1.64E-03**	**0.81**	**3.71**	HCC (Yu et al., 2014a) and others (Gai et al., 2020)
			*MT1E*	−**3.58**	**2.35E-16**	**0.88**	**3.72**	HCC (Liu et al., 2020) and others (Hur et al., 2016)
			*MT1F*	−**3.70**	**3.24E-17**	**0.91**	**3.75**	HCC (Lu et al., 2003) and others (Lin et al., 2017a)
			*MT1G*	−**3.43**	**6.06E-10**	**0.90**	**3.67**	HCC (Wang et al., 2019) and others (Fu et al., 2013)
			*MT1H*	−**2.13**	**1.48E-03**	**0.86**	**2.43**	HCC (Zheng et al., 2017)
			*MT1JP*	−**5.27**	**1.36E-20**	**0.82**	**3.64**	HCC (Wu et al., 2020a) and others (Zhang et al., 2018; Yu et al., 2019)
			*MT1L*	−2.58	5.28E-10	0.86	2.86	Unknown
			*MT1M*	−**3.65**	**2.33E-12**	**0.90**	**2.78**	HCC (Fu et al., 2017) and others (Li et al., 2021)
			*MT1X*	−**3.55**	**1.47E-17**	**0.88**	**3.86**	HCC (Liu et al., 2018b)
			*MT2A*	−3.35	4.85E-24	0.91	3.83	Breast cancer (Kim et al., 2011) and others (Pan et al., 2013)
chr17: 81740217–81874724 (∼134 kb, 14 DEEs)	1.05	4.53	*HGS*	**1.04**	**1.95E-20**	**0.87**	**1.22**	HCC (Canal et al., 2015)
			*CCDC137*	0.95	3.37E-16	0.80	1.78	Unknown
			*NPLOC4*	0.82	3.53E-11	0.79	1.22	Bladder cancer (Lu et al., 2019) and others (Skrott et al., 2017; Pan et al., 2021)
			*CEP131*	**1.42**	**2.39E-19**	**0.79**	**1.78**	HCC (Liu et al., 2017) and others (Kim et al., 2019; Wang et al., 2020)
			*CSNK1D*	0.55	8.36E-08	0.76	1.22	Breast cancer (Bar et al., 2018) and others (Peer et al., 2021)
			*MAFG*	**0.98**	**3.10E-07**	**0.71**	**1.00**	HCC (Liu et al., 2018c)
			*MAFG-DT*	**3.03**	**3.42E-28**	**0.73**	**1.33**	HCC (Ouyang et al., 2019) and others (Cui et al., 2018; Li et al., 2019; Sui et al., 2019; Qu and Liu, 2020; Xiao et al., 2020)
			*FOXK2*	**0.68**	**3.22E-07**	**0.73**	**1.67**	HCC (Lin et al., 2017b) and others (Shan et al., 2016; Nestal de Moraes et al., 2019)
			*SIRT7*	**0.61**	**8.43E-06**	**0.72**	**1.00**	HCC (Zhao et al., 2019) and others (Yu et al., 2014b; Zhang et al., 2015)
			*NARF*	1.07	9.12E-15	0.74	2.00	Glioblastoma (Anderson et al., 2010)

Notes: Enhancer cluster: the cluster of intergenic DEEs that were simultaneously associated with the corresponding cluster of genes; average LFC.DEE: the arithmetic mean of log_2_ fold change of the FPM of all DEEs in the enhancer cluster; average LogFDR.DEE: the arithmetic mean of the–log_10_FDR of the differential expression test of all DEEs in the enhancer cluster; average rho: the arithmetic mean of Spearman correlation coefficients of all DEE-DEG pairs between corresponding DEGs and DEEs in the enhancer cluster; average concurrence: the arithmetic mean of the concurrence of all DEE-DEG pairs between corresponding DEGs and DEEs in the enhancer cluster; implicated cancers: results of literature searching (only molecular mechanism studies) to determine the relevance between DEE-DEG and cancers (genes implicated in HCC were highlighted with a bold font).

Moreover, the identified intergenic DEEs and DEE-DEGs were overall highly replicated in four independent GEO datasets (Table 3). A total of 83.03% of activated DEEs, and 91.33% of repressed DEEs were observed in at least one GEO dataset (i.e., concurrence ≥ 2) (Figure 3A). Furthermore, 54.85% of upregulated DEE-DEGs and 85.01% of downregulated DEE-DEGs were identified in at least one GEO dataset (Figure 3B). Compared with activated DEEs and upregulated DEE-DEGs, those repressed DEEs and downregulated DEE-DEGs were more likely to be conserved in multiple GEO datasets (i.e., concurrence higher than three) (Figures 3A,B). For instance, 54.52% of repressed DEEs and 41.86% of downregulated DEE-DEGs were consistently replicated in more than three GEO datasets, while only 25.34% activated DEEs and 13.71% upregulated DEE-DEGs were also observed in three or more GEO datasets (Figures 3A,B).

**TABLE 3:** Characteristics of five RNA-seq datasets used in the present study.

Dataset	No. of tumor tissues	No. of adjacent tissues	Additional data type	Reference (PMID)
Discovery cohort	33	33	WGBS	—
GSE77314	50	50	—	27119355 (Liu et al., 2016)
GSE124535	35	35	—	30814741 (Jiang et al., 2019)
GSE148355	62	47	—	33772139 (Yoon et al., 2021)
GSE77509	20	20	—	28194035 (Yang et al., 2017)

### Potential Roles of Epigenetic Modification Aberrations in Identified Aberrant Intergenic Enhancers

**FIGURE 4:**
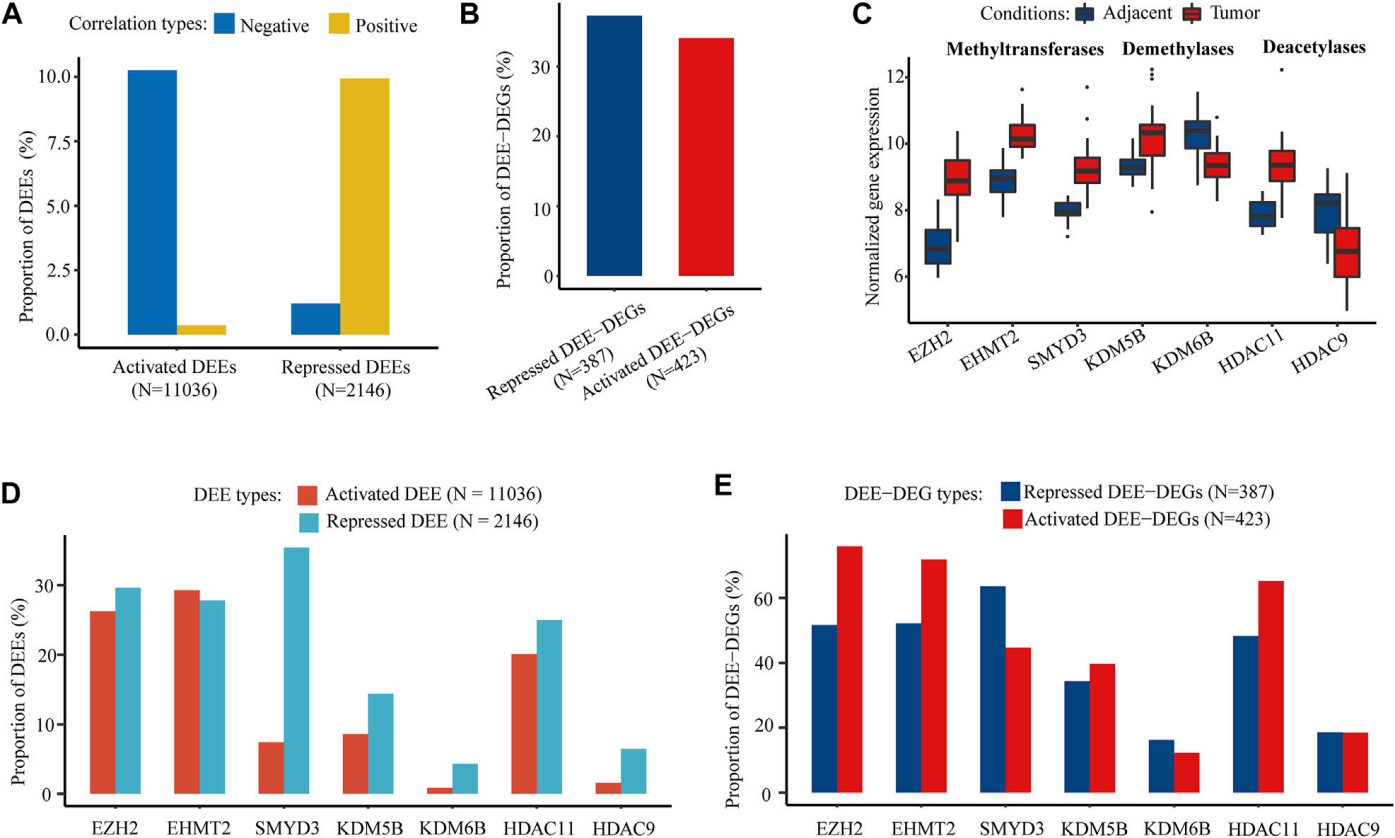
DNA methylation and histone PTM modifer-associated intergenic DEEs and DEE-DEGs. **(A)** Percentage of DNA methylation-associated intergenic DEEs. **(B)** Percentage of DNA methylation-associated intergenic DEE-DEGs. **(C)** Significant differential expression of seven histone modification regulators (three histone methyltransferases, two histone demethylases, and three histone deacetylases) in the discovery cohort. **(D)** Proportion of histone modification regulator-associated intergenic DEEs. **(E)** Percentage of histone modification regulator-associated intergenic DEE-DEGs.

Through integration with matched WGBS data in the discovery cohort, the differential expression of 10.61% of the activated DEEs and 11.14% of the repressed DEEs was significantly correlated with regional differential DNA methylation, especially hypomethylation, in corresponding enhancers (Figure 4A). Nevertheless, these differential methylation-associated DEEs correlated with 34.04% of the upregulated DEE-DEGs and 37.21% of the downregulated DEE-DEGs (Figure 4B), suggesting that those methylation-associated DEEs were more likely to be transcriptional master regulators that targeted multiple genes. In addition to DNA methylation, there was also substantial dysregulation of histone modification in HCC. Three histone methyltransferases (EZH2, EHMT2, and SMYD3), two demethylases (KDM5B and KDM6B), and two deacetylases (HDAC11 and HDAC9) were differentially expressed in both the discovery and four GEO datasets (Figure 4C; Supplementary Table S2.2). Coexpression tests showed that many DEEs and DEE-DEGs were significantly correlated with the differential expression of those seven histone modification regulators, especially EZH2, EHMT2, and SMYD3 (Figures 4D,E). Notably, 75.89% of the upregulated DEE-DEGs and 63.57% of the downregulated DEE-DEGs displayed significant coexpression with EZH2 and SMYD3, respectively, which were much higher than the corresponding percentages for significantly correlated DEEs (26.26 and 35.41%, respectively) (Figures 4D,E), suggesting that histone modification-associated DEEs are also more likely to be transcriptional master regulators.

### Aberrant Genic Enhancers-Associated With DNA Methylation Alterations

**FIGURE 5:**
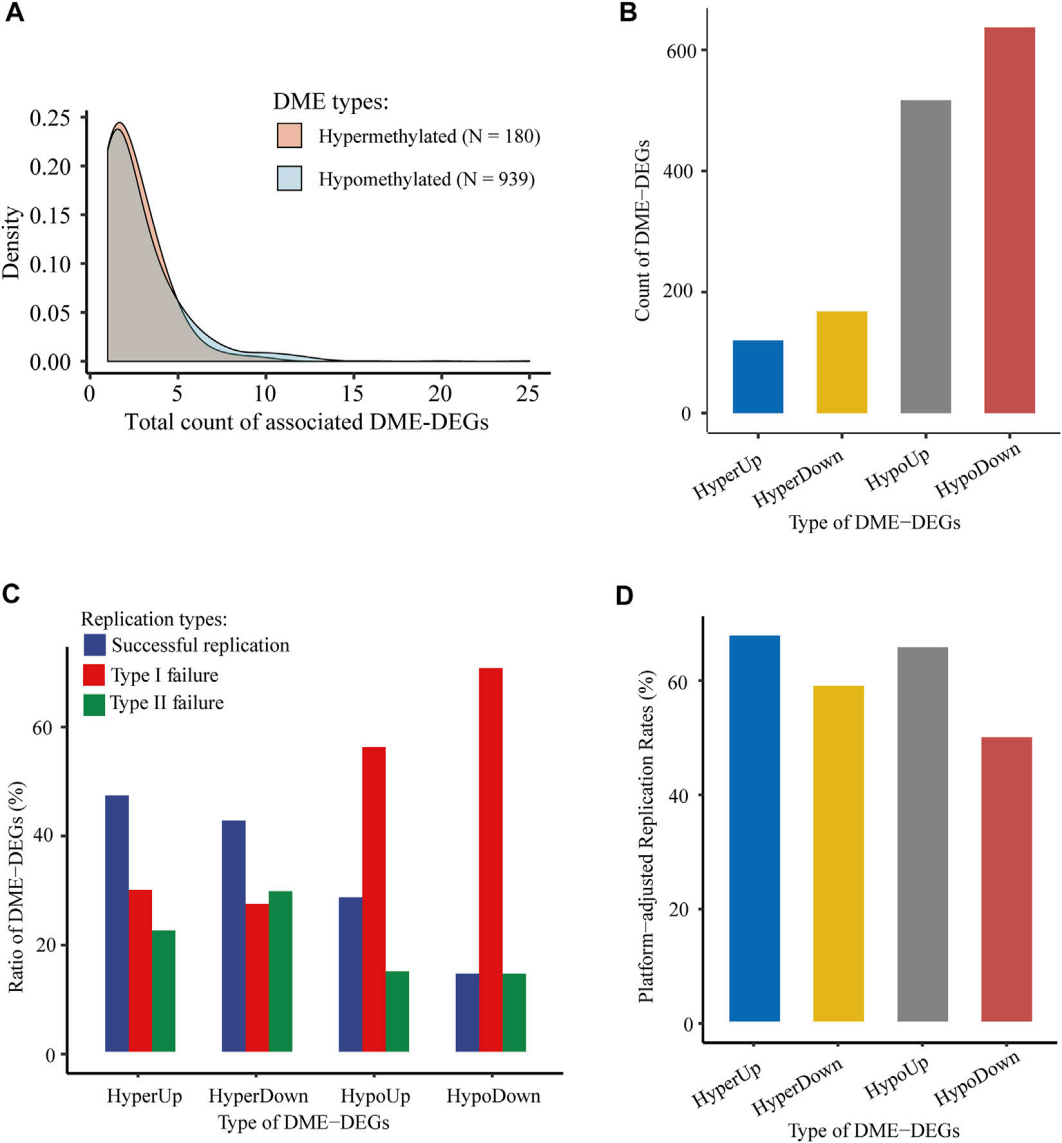
Identification and validation of genic DMEs and associated DME-DEGs in HCC. **(A)** Distribution density of the number of associated DME-DEGs of hypermethylated and hypomethylated genic DME. **(B)** Count of four types of genic DME-DEGs. “HyperUp” refers to hypermethylated enhancer-associated upregulated DME-DEGs; “HyperDown” refers to hypomethylated enhancer-associated downregulated DME-DEGs; “HypoUp” refers to hypermethylated enhancer-associated upregulated DME-DEGs, and “HypoDown” refers to hypomethylated enhancer-associated downregulated DME-DEGs. **(C)** Distribution of the three types of replication results of genic DME-DEGs. “Successful replication” refers to the successful replication of genic DME-DEGs for correlated differential methylation and differential expression in TCGA-LIHC; “type I failure” refers to replication failure due to lack of CpG for the corresponding genic DMEs in TCGA-LIHC; and “type II failure” refers to replication failures except type I failure. **(D)** Platform-adjusted replication rates of four types of genic DME-DEGs in TCGA-LIHC. Platform-adjusted replication rates were calculated as (Count_Successful Replication_ + Count_Successful Replication_ + Count_Type I Failure_) * 100%.

Among those collected genic enhancers, 1,119 DMEs and their associated DME-DEGs were identified through the integration of WGBS, ChIP-seq, and RNA-seq data. Overall, hypomethylated and hypermethylated DMEs displayed similar transcriptional regulation patterns (i.e., they tended to be correlated with equal number of DME-DEGs) (Figure 5A). In total, there were 1,442 genic DEE-DEGs, including 120 hypermethylated upregulated DEE-DEGs (HyperUp), 168 hypermethylated downregulated DEE-DEGs (HyperDown), 517 hypomethylated upregulated DEE-DEGs (HypoUp), and 637 hypomethylated downregulated DEE-DEGs (HypoDown) (Figure 5B; Supplementary Table S3.1). Approximately half (52.50%) of the identified DEE-DEGs exhibited a nonclassical positive correlation between DNA methylation and gene expression. The results of independent replication of those 1,442 DME-DEGs in TCGA-LIHC showed that the raw replication rates of the four types of DEE-DEGs (i.e., HyperUp, HyperDown, HypoUp, and HypoDown) were 47.50, 42.86, 28.63, and 14.44%, respectively (Figure 5C). Since the 450 k methylation array barely covered CpGs located in the gene body and intergenic regions, which were primarily hypomethylated, it was not surprising to observe much lower raw replication rates and higher type I failure ratios for the HypoUp and HypoDown groups. In contrast, their platform-adjusted replication rates reached 67.86, 59.02, 65.78, and 50.00% (Figure 5D), which were comparable to each other.

### Intergration of Aberrant Enhancer-Associated Transcriptional Dysregulation and Sucessfully in Silico Verification Based on HiC Data

**FIGURE 6:**
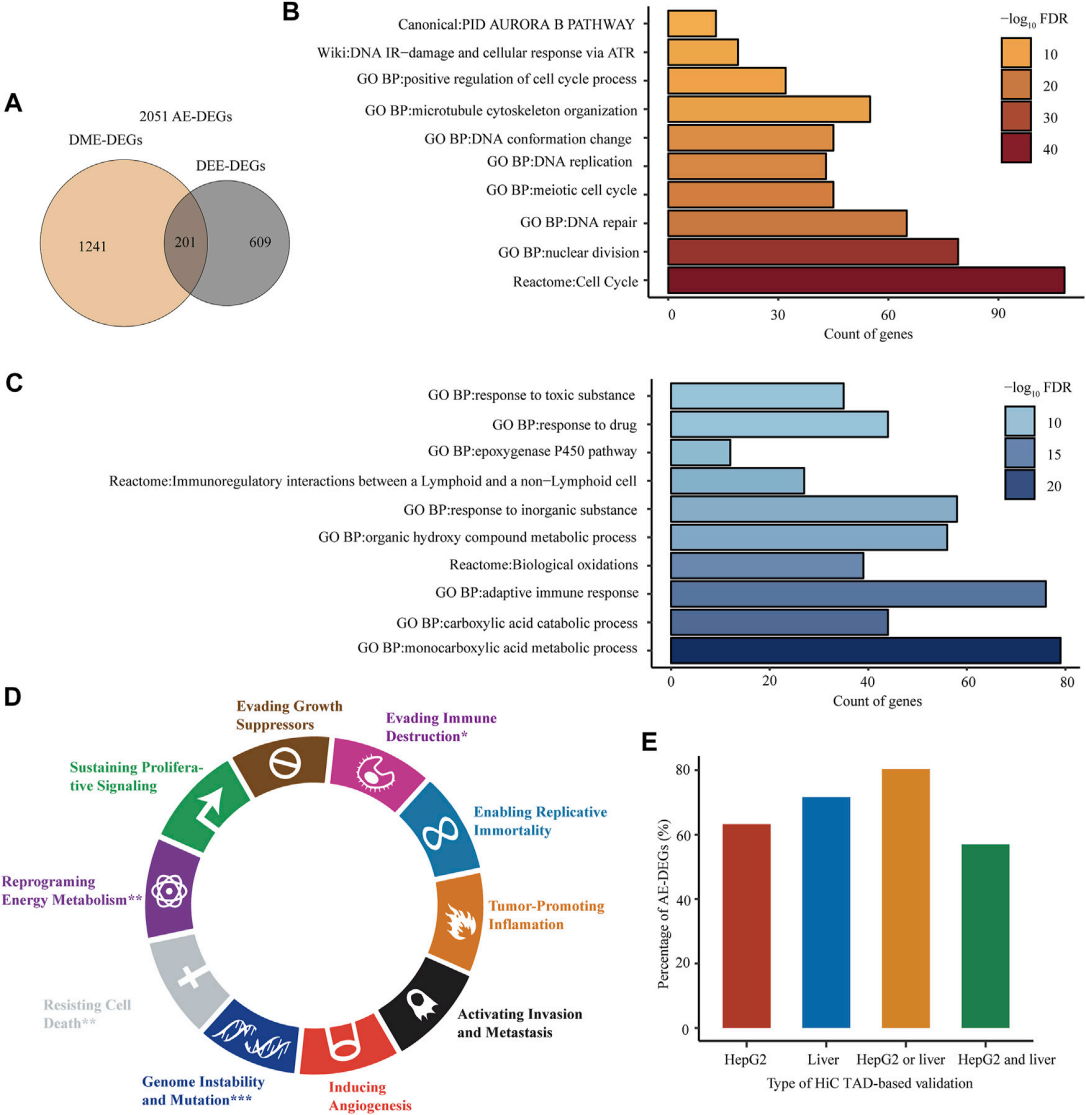
Biological functions and in silico verification of AE-DEGs. **(A)** Venn diagram displaying the overlap between genic DME-DEGs and intergenic DEE-DEGs. The union of them were defined as aberrant enhancer-associated DEGs (AE-DEGs). **(B)** and **(C)** Top ten overrepresented pathways/GO terms of activated AE-DEGs and repressed AE-DEGs, respectively. **(D)** Enrichment of AE-DEGs for ten cancer hallmarks. “*”refers to hypergeometric test FDR < 0.05; “**”refers to FDR < 1e-2; and “***”refers to FDR < 1e-3. **(E)** Percentage of AE-DEGs that were successfully validated by TADs in HiC-profiled HepG2 and normal liver samples. “HepG2 or liver” refers to successful validation in HepG2 or the liver sample; “HepG2 and liver” refers to successful validation in both HepG2 and the liver sample.

After combining 1,442 genic DME-DEGs with the 810 intergenic DEE-DEGs, we obtained a set of 2,051 aberrant enhancer-associated DEGs (AE-DEGs), which was composed of 1,092 upregulated AE-DEGs and 959 downregulated AE-DEGs (Figure 6A; Supplementary Table S3.2). Pathway/biological process enrichment analyses demonstrated that 1,092 activated AE-DEGs were overrepresented for genes implicated in the cell cycle, nuclear division, DNA repair, and DNA replication (Figure 6B), while 959 repressed AE-DEGs were enriched for genes involved in monocarboxylic acid metabolism, adaptive immune response, biological oxidation, and cytochrome P450 epoxygenase pathway (Figure 6C). Moreover, hypergeometric test revealed that AE-DEGs showed significant enrichment for genes related to four cancer hallmarks including genome instability and mutation (FDR = 6.3e−9), reprogramming energy metabolism (FDR = 1.2e−3), resisting cell death (FDR = 7.3e−3), and evading immune destruction (FDR = 4.2e−2) (Figure 6D).

In addition, AE-DEGs were further verificated according to HiC-produced TADs and chromatin loops. The results showed that 63.24 and 71.62% of AE-DEGs were in the same TAD, in which their corresponding enhancers were located, in the HiC profiled HepG2 and normal adult liver tissue sample (Figure 6E; Supplementary Table S3.3). Moreover, the TAD validation results in two HiC samples were highly consistent. Specifically, 56.95 and 80.35% of AE-DEGs were successfully supported by TADs in both samples and either sample, respectively (Figure 6E; Supplementary Table S3.3). Furthermore, 12 and one AE-DEG were confirmed by chromatin interaction loops in HepG2 and normal liver sample (Supplementary Table S3.4), respectively.

### Construction of a Six AE-DEGs -Based Prognostic Model

**FIGURE 7:**
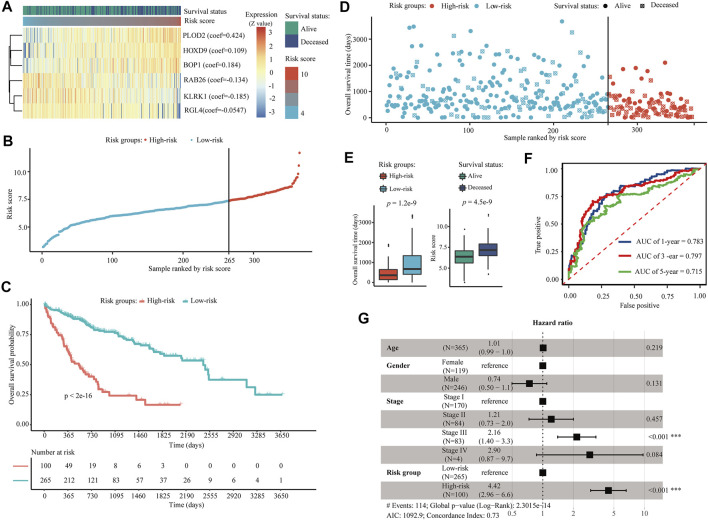
Construction of a six AE-DEG-based prognostic model for HCC in TCGA-LIHC. **(A)** The expression heatmap of six AE-DEGs constituted the identified prognostic model for OS of HCC in TCGA-LIHC. Multivariate Cox regression derived coefficients used for the calculation of risk score are given in parentheses. Patients were ranked according to corresponding calculated risk scores. **(B)** Distribution of the calculated risk scores of HCC patients in TCGA-LIHC. **(C)** Kaplan-Meier analysis of the six AE-DEG-based prognostic signature in TCGA-LIHC. **(D)** Distribution of duration and survival status of HCC patients in TCGA-LIHC. **(E)** Box plots display the comparison of survival times between high- and low-risk HCC patients and the comparison of risk scores between alive and deceased HCC patients in TCGA-LIHC. Wilcox *p*-values were calculated and displayed with each boxplot. **(F)** Time-dependent ROC analyses of the six AE-DEG-based prognostic signature in TCGA-LIHC. **(G)** Forest plot of the multivariate Cox regression analysis in TCGA-LIHC.

Through univariate Cox regression, LASSO, and multivariate Cox regression model filtering, 2051 AE-DEGs were eventually filtered to six genes to build a prognostic model for OS in TCGA-LIHC. These six AE-DEGs included procollagen-lysine,2-oxoglutarate 5-dioxygenase 2 (PLOD2), homeobox D9 (HOXD9), BOP1 ribosomal biogenesis factor, which is also known as Block of Proliferation (BOP1), Ras-related protein Rab-26 (RAB26), killer cell lectin-like receptor K1 (KLRK1), and Ral guanine nucleotide dissociation stimulator like 4 (RGL4) (Figure 7A). A prognostic risk score was calculated for each patient as follows: the risk score = (0.424 * expression of PLOD2) + (0.109 * expression of HOXD9) + (0.184 * expression of BOP1) + (−0.134 * expression of RAB26) + (−0.185 * expression of KLRK1) + (−0.0547 * expression of RGL4). An optimal cutoff at 7.37 was applied to divide all patients into high-risk (N = 100) and low-risk (N = 265) groups (Figure 7B). Kaplan-Meier analysis revealed significant differences in OS probability across time between high-risk and low-risk groups (*p* < 2.0e−16) (Figure 7C). Wilcox rank-sum exact tests illuminated significantly less OS duration in high-risk patients (*p* = 1.2e−9), and lower risk scores among alive patients (*p* = 4.5e−9) (Figures 7D,E). The areas under the time-dependent ROC curves (AUCs) for 1-, 3-, and 5-years OS were estimated to be 0.783, 0.797, and 0.715, respectively (Figure 7F). A multivariate Cox regression model constructed using both age, gender, and pathologic TNM stage demonstrated that TNM stage (*p* < 0.001, HR = 2.16) and risk group (*p* < 0.001, HR = 4.42) were both independent prognostic biomarkers for OS of HCC patients in TCGA-LIHC (Figure 7G).

**FIGURE 8:**
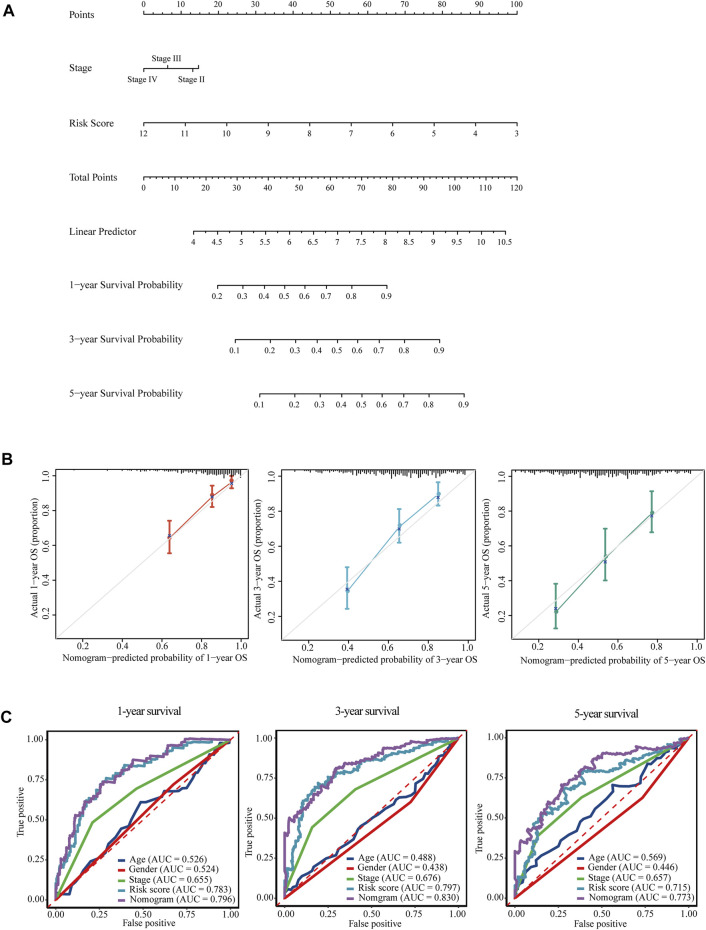
Nomogram for the prediction of overall survival of HCC in TCGA-LIHC. **(A)** A prognostic nomogram for predicting the probabilities of 1-year, 3-years, and 5-years overall survival of HCC patients in TCGA-LIHC. **(B)** Calibration plots for evaluation of the predictive performance of the constructed nomogram. **(C)** Time-dependent ROC curves displayed the comparisons of AUCs among diverse prognostic models.

Subsequently, a predictive nomogram was built by combining the risk score and TNM stage for accurate prediction of overall survival probability in 1, 3, and 5 years (Figure 8A). The calibration plots for internal validation of the nomogram showed high consistency between the predicted OS outcomes and actual observations (Figure 8B). Time-dependent ROC curves revealed the best predictive performance of the nomogram, with AUCs of 0.796, 0.830, and 0.773 for 1-year, 3-years, and 5-years OS, respectively (Figure 8C).

### Consistent Validation of the Six AE-DEGs-Based Prognostic Model in ICGC-LIRI

**FIGURE 9:**
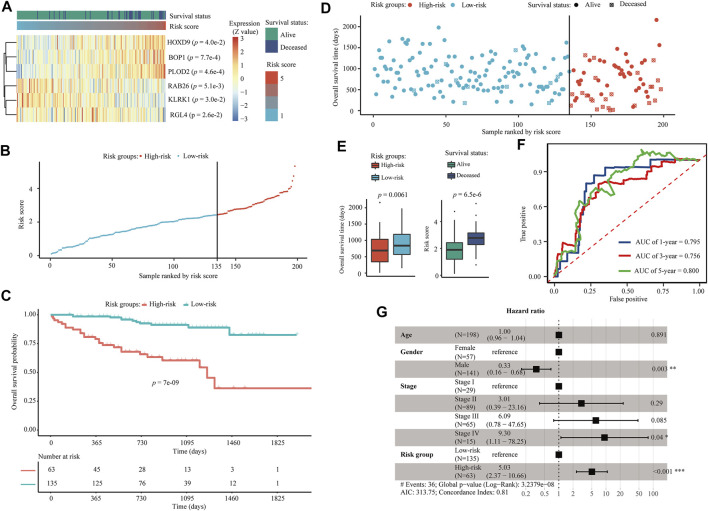
Validation of the six AE-DEG-based prognostic model for OS of HCC in ICGC-LIRI cohort. **(A)** The expression heatmap of six AE-DEGs constituted the identified prognostic model for overall survival of HCC in ICGC-LIRI cohort. Patients were ranked according to their risk scores. **(B)** Distribution of the calculated risk scores of HCC patients in ICGC-LIRI. **(C)** Kaplan-Meier analysis of the 6-gene prognostic signature in ICGC-LIRI. **(D)** Distribution of duration and survival status of HCC patients in ICGC-LIRI. **(E)** Boxplots display the comparison of survival time between high- and low-risk HCC patients and the comparison of risk score between alive and deceased HCC patients in ICGC-LIRI. **(F)** Time-dependent ROC analysis of the six AE-DEG-based prognostic signature in ICGC-LIRI. **(G)** Forest plot of the multivariate Cox regression analysis in ICGC-LIRI.

Univariate Cox regression revealed that all six AE-DEGs that constituted the identified prognostic signature were significant OS-related biomarkers in ICGC-LIRI cohort (Figure 9A). Risk scores were calculated for each ICGC-LIRI patient by using the coefficients estimated from TCGA-LIHC. Similarly, 198 ICGC-LIRI patients were divided into high-risk (N = 65) and low-risk (N = 135) groups according to the corresponding optimal cutoff (Figure 9B). Kaplan-Meier analysis revealed significant differences in OS probability across time between high-risk and low-risk group ICGC-LIRI patients (*p* = 7.0e−9) (Figure 9C). Wilcox rank-sum exact tests illuminated significantly less OS duration in high-risk patients (*p* = 0.0061), and lower risk scores among alive patients (*p* = 6.5e−6) (Figures 9D,E). The AUCs for 1-, 3-, and 5-years OS were estimated as 0.795, 0.756, and 0.800 (Figure 9F), respectively. A multivariate Cox regression model constructed using both age, gender, and pathologic TNM stage also confirmed that the risk group (*p* < 0.001, HR = 5.03) was an independent prognostic biomarker for OS of HCC patients in ICGC-LIRI (Figure 9G).

### The Six AE-DEG-Based Prognostic Model Display Superb Predictive Performance for OS of HCC Patients

Furthermore, the good predictive performance of our identified AE-DEG-based signature was assessed through comparisons with seven established similar prognostic models (Long et al., 2019; Zhang et al., 2020b; Ouyang et al., 2020; Tang et al., 2020; Zhu et al., 2020; He et al., 2021; Wang et al., 2021). Among all signatures, the hypoxia-related gene-based signature and our AE-DEG-based signature were the only two models in which all AUCs were higher than 0.7, which is a well-accepted criterion for high predictive accuracy. Moreover, our model’s average AUCs in the discovery and validation cohorts were both higher than those of the hypoxia-related model (0.765 and 0.784 *vs.* 0.723 and 0.763) (Table 4). Overall, our prognostic signature had more predictive power than others.

**TABLE 4:** Comparison of the predictive performance of our AE-DEG-based signature with seven previously established prognostic signatures in HCC.

Signature name	AUCs for OS in discovery			AUCs for OS in validation		
	1-year	3-years	5-years	1-year	3-years	5-years
Methylation-driven gene based signature 1 (Long et al., 2019)	0.6885	0.6563	0.6548	0.6397	0.6644	0.5942
Methylation-driven gene based signature 2 (He et al., 2021)	0.742	0.661	—	0.695	0.655	—
Angiogenic gene based signature (Zhu et al., 2020)	0.74	0.66	0.66	0.78	0.74	—
EMT related gene based signature (Wang et al., 2021)	0.824	0.798	0.800	0.688	0.674	0.876
Ferroptosis and iron-metabolism related gene based signature (Tang et al., 2020)	0.77	0.71	0.64	0.67	0.73	—
Differentially expressed gene signature (Ouyang et al., 2020)	0.77	0.73	0.72	0.63	0.68	0.65
Hypoxia related gene based signature (Zhang et al., 2020b)	0.78	0.70	0.70	0.75	0.77	0.77
Our AE-DEG based signature	0.783	0.797	0.715	0.795	0.756	0.800

## Discussion

In the present study, integration of ChIP-seq and RNA-seq data revealed substantial intergenic DEEs and associated DEE-DEGs in HCC. Compared with activated DEEs and DEE-DEGs, the repressed DEEs and DEE-DEGs displayed higher consistency in multiple HCC cohorts. Remarkably, 162 of those 387 intergenic DEE-DEGs were concurrent in at least four of the five HCC cohorts that were analyzed in this study. Functional enrichment analysis by Metascape (Zhou et al., 2019) revealed that half of those highly concurrent genes were liver-specific. Enrichment of repressed DEGs for liver-specific genes was previously reported in HCC (Lian et al., 2018). A highly plausible mechanism underlying this phenomenon might be cell dedifferentiation. Cell dedifferentiation is a process that implicates the epigenetic reprogramming of gene activity to transform cells into a less differentiated state like their parent cell type. In the development of HCC, stepwise dedifferentiation is a certain event that exhibits loss of hepatic functions and morphology and gain of hepatic progenitor markers (Chao et al., 2020). Moreover, the well-known demethylation agent 5-azacytidine (5-AZA) displayed potential for usage in dedifferentiation therapy in HCC cell lines and cell-derived xenograft (Gailhouste et al., 2018). In addition, it has been shown that upon loss of the mouse Igκ gene’s downstream enhancers, E3′ and Ed, the mature B cells unexpectedly undergo reversible retrograde differentiation (Zhou et al., 2013). Hence, our finds about conservative enhancer repression associated suppression of liver-specific genes might shed new light on epigenetic mechanisms underlying the dedifferentiation that occurs in hepatocarcinogenesis and provide potential targets for dedifferentiation-targeted therapy of HCC.

Notably, highly conserved intergenic DEE-DEGs with counts less than 200 unexpectedly included the majority of all MT genes in the genome. MTs are small cysteine-rich proteins that play pivotal roles in metal homeostasis and protection against heavy metal-related cytotoxicity, DNA damage, and oxidative stress (Coyle et al., 2002). Dysregulation of MTs is ubiquitous in most malignancies, and emerging evidence shows that MTs are implicated in tumor formation, progression, and drug resistance (Si and Lang, 2018; Merlos Rodrigo et al., 2020). As mentioned earlier, nine identified DEE-associated differentially expressed MT isoforms were reported to be involved in liver cancer. Specifically, the upregulation of *MT1A* mediated the attenuation of malignant behaviors of *CT23* knockdown in HCC cells (Ning et al., 2021). *MT1DP* is a pivotal anticancer long noncoding RNA (lncRNA), whose suppression mediates the vital carcinogenetic roles of *RUNX2* and *YAP* in HCC (Yu et al., 2014a). *MT1E* was newly identified as a novel tumor suppressor for HCC that could induce apoptosis and suppress cell growth and metastasis (Liu et al., 2020). Exogenous expression of *MT1F* displayed a strong inhibitive effect on the growth of HepG2 cells (Lu et al., 2003). *MT1G* was uncovered as a tumor suppressor in HCC by inducing the transcriptional activity of p53 through direct interaction and supply of appropriate zinc ions to p53 (Wang et al., 2019). *MT1H* functions as a tumor suppressor that suppresses the proliferation and invasion of HCC cells by inhibiting the Wnt/β-catenin pathway (Zheng et al., 2017). Overexpression of the lncRNA *MT1JP* remarkably inhibited the proliferation and enhanced apoptosis, which might be mediated by regulating the expression of *AKT* (Wu et al., 2020a). Similarly, *MT1M* also showed a tumor-suppressive ability to suppress cell viability, migration, and invasion and activate apoptosis *in vitro* (Fu et al., 2017). *MT1X* was demonstrated to be a tumor suppressor that suppresses tumor growth and metastasis *in vivo* and induces cell cycle arrest and apoptosis by repressing the NF-κB signaling pathway in HCC (Liu et al., 2018b). The roles of *MT1CP*, *MT1L*, and *MT2A* in HCC are still unknown, while *MT2A* could promote breast cancer invasiveness and might play a suppressive role in gastric cancer through inhibition of the NK-κB signaling pathway (Kim et al., 2011; Pan et al., 2013).

Besides, there were also several sets of upregulated genes associated with activated super-enhancers in HCC. On chromosome 17, a group of 10 activated DEE-DEGs was found to be associated with increased enhancer activity for 14 intergenic DEEs. It is intriguing that six (*HGS*, *CEP131*, *MAFG*, *MAFG-DT*, *FOXK2*, and *SIRT7*) of them have already been discovered as proto-oncogenes in HCC (Canal et al., 2015; Lin et al., 2017b; Liu et al., 2017; Liu et al., 2018c; Ouyang et al., 2019; Zhao et al., 2019). In particular, the lncRNA *MAFG-DT*, which is likewise known as *MAFG-AS1*, was also recently shown to play oncogenic roles in multiple tumors in addition to HCC, including colorectal cancer (Cui et al., 2018), breast carcinoma (Li et al., 2019), bladder urothelial carcinoma (Xiao et al., 2020), esophageal squamous cell carcinoma (Qu and Liu, 2020), and lung adenocarcinoma (Sui et al., 2019). *NPLOC4*, also known as *NPL4*, is uncharacterized in HCC but has been revealed as an important oncogene in bladder cancer (Lu et al., 2019) and a critical target of the anticancer drug disulfiram (Skrott et al., 2017; Pan et al., 2021). Similarly, *CSNK1D* has recently been identified as a novel drug target in Hedgehog/GLI-driven cancers (Peer et al., 2021), and silencing of *CSNK1D* attenuates the migration and metastasis of triple-negative breast cancer cells (Bar et al., 2018). As an E3 ubiquitin ligase, *NARF* was identified as a positive regulator of cell growth in glioblastoma (Anderson et al., 2010). *CCDC137* has not been characterized in any cancer, but its depletion via HIV could cause cell cycle arrest (Zhang and Bieniasz, 2020). Taken together, the elevated activity of the super-enhancer, which is composed of a cluster of 14 synergistic enhancers located on chromosome 17, was demonstrated to be associated with the activation of several critical oncogenes implicated in HCC and/or other cancers. Therefore, inhibition of this activated super-enhancer might be a promising therapy for HCC.

Our integrative transcriptomic analyses discovered massive concurrent DEEs in HCC, which might be caused by either genetic mutations or epigenetic aberrations. However, those DEEs, especially repressed DEEs, were ubiquitous and conserved in multiple HCC cohorts, which suggests a higher possibility of epigenetic aberration-relevant underlying mechanisms. Indeed, our investigation revealed that considerable DEEs and DEE-DEGs were linked to DNA methylation and histone modification. Notably, there were strong associations between the activation of three histone methyltransferases (*EZH2*, *EHMT2*, and *SMYD3*) and enhancer aberrations. This was consistent with the previous findings that mutations and expression changes of epigenetic modifiers are common events leading to an aggressive gene expression and poor clinical outcomes in HCC (Bayo et al., 2019). *EZH2*, *EHMT2*, and *SMYD3* are vital epigenetic regulators that could be targeted for cancer therapy (Cheng et al., 2019). Unlike those of *EZH2* (Gao et al., 2014; Liu et al., 2015; Zhuang et al., 2016; Chen et al., 2018b), the roles of *EHMT2* and *SMYD3* in mediating transcriptional regulation in carcinogenesis are still not fully characterized in HCC (Zhang et al., 2021a; Guo et al., 2021). Our findings serve as a proof-of-concept that activation of histone methyltransferases, such as *EZH2*, *EHMT2*, and *SMYD3* might promote hepatocarcinogenesis by inducing enhancer aberration of crucial cancer-related genes.

To better assess the clinical outcomes of HCC patients, in this study, we applied machine learning approaches to explore the prognostic significance of AE-DEGs in HCC and established a prognostic model based on a panel of six AE-DEGs, including *PLOD2*, *HOXD9*, *BOP1*, *RAB26*, *KLRK1*, and *RGL4*. Our identified AE-DEG-based signature outperformed clinical characteristics such as the TNM stage and seven previously established similar prognostic models in terms of predictive accuracy, suggesting that those six AE-DEGs might play important roles in HCC. *PLOD2* encodes a key enzyme mediating the formation of the stabilized collagen cross-links, which are considered as the “highway” for cancer cell migration and invasion (Provenzano et al., 2006). The roles of *PLOD2* in breast cancer, sarcoma, bladder cancer, and renal cell carcinoma were thoroughly discussed in a previous review (Du et al., 2017). *PLOD2* was first demonstrated as a prognostic marker for HCC in 2011 (Noda et al., 2012), while the function and mechanism of *PLOD2* activation in HCC have not been thoroughly explored. *HOXD9* and *BOP1* were both uncovered as the oncogenic promoters of epithelial-mesenchymal transition (EMT) in HCC (Chung et al., 2011; Lv et al., 2015), which was in line with their unfavorable prognostic contribution in our identified prognostic signature. On the other hand, *RAB26* was novel in HCC but was newly identified as a suppressor of the migration and invasion of breast cancer cells (Liu et al., 2021). The roles of *KLRK1* and *RGL4* have not been investigated in any malignancies but have been identified as prognostic factors in lung adenocarcinoma (Sun et al., 2020; Zhang et al., 2021b). In summary, previous studies revealed pivotal cancer-related functions of *PLOD2*, *HOXD9*, *BOP1*, and *RAB26,* manifesting our findings of their AE-associated dysregulation and prognostic significance in OS of HCC, and suggesting the possibility that *PLOD2*, *RAB26*, *KLRK1*, and *RGL4* play essential roles in the progression and survival of HCC, although further experimental investigations are warranted.

Our study uncovered systematic enhancer aberrations with important functions and excellent prognostic significance in HCC. There are still several potential limitations. First of all, RNA-seq is still commonly used in the literature (Chen et al., 2018a; Wu et al., 2020b; Chen and Liang, 2020) but is not one of the best choices for the comprehensive detection of eRNA; for example, GRO-seq would be a better approach (Danko et al., 2015; Zhang et al., 2020c). Second, aberrant genic enhancers might be only partially captured by identifying genic DMEs, especially considering the relatively low ratio of DNA methylation-associated DEEs in total intergenic DEEs. Moreover, although our identified AE-DEGs were successfully replicated in independent cohorts and confirmed by TADs from HiC, further validation of enhancer-mediated transcriptional regulation of particular genes *via* experimental technologies such as CRISPR, like in previous enhancer related studies (Chen et al., 2018a; Xiong et al., 2019), was lacking in our present study and will be part of our ongoing works.

## Conclusion

In conclusion, our integrative analysis of the epigenome and transcriptome depicted and verified a systematic landscape of aberrant enhancers and 2051 associated DEGs, including many well-known cancer-related genes, in HCC. These findings provide new insight into the roles of epigenetic aberration induced aberrant enhancers in the progression of HCC. Furthermore, our established prognostic signature based on six AE-DEGs displayed superior predictive performance over previous models for predicting the long-term and short-term OS of HCC patients.

## Data Availability

The datasets presented in this study can be found in online repositories. The names of the repository/repositories and accession number(s) can be found below: NCBI SRA database [accession: PRJNA762641].
