# Supplementary material for: Integrating the Epigenome and Transcriptome of Hepatocellular Carcinoma to Identify Systematic Enhancer Aberrations and Establish an Aberrant Enhancer-Related Prognostic Signature
Source: Front Cell Dev Biol. 2022 Mar 1;10:827657. doi: 10.3389/fcell.2022.827657 (PMC8921559; doi:10.3389/fcell.2022.827657)
Supplement: Supplementary file 1 [file DataSheet1.ZIP › Supplementary methods.docx]

**Supplementary methods**

1. **High-Throughput Sequencing**

Paired tumor and adjacent non-tumor tissue samples from 33 HCC patients were subjected to whole-genome bisulfite sequencing on the Illumina X Ten platform with the procedures described in our previous WGBS paper [1]. Briefly, a 200-ng genomic DNA sample was sheared to about 300-bp fragments by sonication. Then DNA fragments were subjected to end-repair, addition of adenosine to the 3′ end, and TruSeq adaptor ligation (Illumina, San Diego, CA USA). Bisulfite conversion was implemented via the EZ DNA methylation kit (Zymo Research, Irvine, CA USA) according to the manufacture’s protocol. After that, bisulfite-converted DNA was enriched through several cycles of PCR amplification using the KAPA HiFi HotStart uracil DNA polymerases (Kapa Biosystems, Boston, MA USA). The PCR conditions were set as 45 s at 98°C followed by 10 cycles at 98°C for 15 s, 65°C for 30 s, 72°C for 30 s, ending with 72°C for 1 min. The quality of each WGBS library was assessed by Qubit 2.0 (Life Tech, Carlsbad, CA USA) and an Agilent 2100 Bioanalyzer. Finally, 150-bp pair-end sequencing was conducted on the Illumina X Ten sequencing platform. High-throughput mRNA-seq was performed for each WGBS sample. Similarly, all 66 RNA samples with high-quality (RIN ≥ 7) were applied to the Illumina X Ten platform for sequencing. Specifically, total RNA was extracted and purified using the RNeasy Micro Kit (Qiagen, Valencia, CA USA) according to the manufacturer’s instructions. The quality of RNA was assessed via an Agilent 2100 Bioanalyzer. Libraries for poly(A)+ RNA were prepared according to the Illumina standard protocol. Constructed libraries were sequenced on HiSeqX Ten platform by Wuxi AppTec (Wuxi, Jiangsu China).

1. **Quality Control of WGBS and RNA-seq Data**

For raw reads from RNA-seq, Cutadapter [2] (v.1.12) and Trimmomatic [3] (v. 0.33) were applied for adapter removal and trimming of low-quality sequences, followed by FastQC (http://www.bioinformatics.babraham.ac.uk/projects/fastqc) for a quality check. The clean WGBS reads that passed similar preprocessing were then aligned with the hg38 reference genome using Bismark (v. 0.16.1) [10] with default parameters. As recommended by the R package DSS (v.2.26.0) developer [11], the smoothing approach was adopted for estimation of smoothed methylation level for all 28.9 million CpGs with default parameters.

1. **Identification of Differentially Methylated Loci (DML)**

In order to identify overall significant differential methylation between all tumor and non-tumor samples, a combined Baumgartner–Weiβ–Schindler (BWS) test [12] was applied to carry out age-adjusted DML detection via the R package BWStest (v.0.2.2). We divided all the 33 HCC patients into three age groups: “young” (age < 55 years; n = 10), “medium” (55 < age ≤ 65; n = 13), and “old” (age > 65; n = 10). A single BWS test was performed for each age group on every CpG to obtain two individual BWS *p*-values (*p*_left_ and *p*_right_). Afterward, three one-sided *p*-values were combined as statistic T_left_ (or T_right_) = -2* Σlog_10_(*p*_left_ [or *p*_right_]), and a new statistic T was defined as max(T_left_, T_right_)[12]. The empirical distribution of the T statistics of combined BWS test was determined by 2.0 × 10^8^ time permutations. At last, an overall empirical *p*-value was estimated as the combined BWS *p*-values for each CpG. CpG with a combined BWS *p*-value < 1.0 × 10^-5^ was identified as DML for subsequent DMR calling.

1. **Calling of Differentially Methylated Regions (DMRs)**

Tumor-associated DMRs were determined by R script with the following two steps: 1) DML were combined into pre-DMRs if the distance between neighbor CpGs was < 200 bp; and 2) all CpGs located between the start and the end of each pre-DMR were included as a final DMR. The arithmetic mean of T statistics for all CpGs in each DMR was calculated for estimating the empirical combined BWS *p*-value for each DMR. Group-level methylation was estimated as the arithmetic average of DNA methylation of all CpGs in each corresponding DMR.

Reference

1. Wang M, Zhao J, Wang Y, Mao Y, Zhao X, Huang P, Liu Q, Ma Y, Yao Y, Yang Z *et al*: **Genome-wide DNA methylation analysis reveals significant impact of long-term ambient air pollution exposure on biological functions related to mitochondria and immune response**. *Environmental Pollution (Barking, Essex: 1987)* 2020, **264**:114707.

2. Martin M: **Cutadapt removes adapter sequences from high-throughput sequencing reads**. *EMBnet journal* 2011, **17**(1):pp. 10-12.

3. Bolger AM, Lohse M, Usadel B: **Trimmomatic: a flexible trimmer for Illumina sequence data**. *Bioinformatics* 2014, **30**(15):2114-2120.

4. Bray NL, Pimentel H, Melsted P, Pachter L: **Near-optimal probabilistic RNA-seq quantification**. *Nature biotechnology* 2016, **34**(5):525-527.

5. Harrow J, Frankish A, Gonzalez JM, Tapanari E, Diekhans M, Kokocinski F, Aken BL, Barrell D, Zadissa A, Searle S *et al*: **GENCODE: the reference human genome annotation for The ENCODE Project**. *Genome research* 2012, **22**(9):1760-1774.

6. Soneson C, Love MI, Robinson MD: **Differential analyses for RNA-seq: transcript-level estimates improve gene-level inferences**. *F1000Res* 2015, **4**:1521.

7. Dobin A, Davis CA, Schlesinger F, Drenkow J, Zaleski C, Jha S, Batut P, Chaisson M, Gingeras TR: **STAR: ultrafast universal RNA-seq aligner**. *Bioinformatics* 2013, **29**(1):15-21.

8. Quinlan AR, Hall IM: **BEDTools: a flexible suite of utilities for comparing genomic features**. *Bioinformatics* 2010, **26**(6):841-842.

9. Love MI, Huber W, Anders S: **Moderated estimation of fold change and dispersion for RNA-seq data with DESeq2**. *Genome biology* 2014, **15**(12):550.

10. Krueger F, Andrews SR: **Bismark: a flexible aligner and methylation caller for Bisulfite-Seq applications**. *Bioinformatics* 2011, **27**(11):1571-1572.

11. Park Y, Wu H: **Differential methylation analysis for BS-seq data under general experimental design**. *Bioinformatics* 2016, **32**(10):1446-1453.

12. Huang H, Chen Z, Huang X: **Age-adjusted nonparametric detection of differential DNA methylation with case-control designs**. *BMC bioinformatics* 2013, **14**:86.
